# Challenges and perception of communal farmers on cattle production in Ga- Matlala, Limpopo Province, South Africa

**DOI:** 10.1016/j.heliyon.2023.e14190

**Published:** 2023-03-05

**Authors:** Monkwe TR, Gxasheka M, Gunya B

**Affiliations:** a Department of Agricultural Economics and Animal Production, School of Agricultural and Environmental Sciences, Faculty of Science and Agriculture, University of Limpopo, Sovenga, 0727, South Africa; b Department of Plant Production, Soil Science & Agricultural Engineering, School of Agricultural and Environmental Sciences, Faculty of Science and Agriculture, University of Limpopo, Sovenga, 0727, South Africa

**Keywords:** Communal farmers, Cattle, Management practices

## Abstract

A survey was carried out to assess challenges and communal farmers' views on cattle production. In Ga-Matlala, 59 community cattle farmers from three rural villages were interviewed (Phofu, Phetole, and Madietane). The majority of responders (49.2%) were from Madietane, with an equal number (25.4%) from Phetole and Phofu (25.4%). In all three villages studied, males outnumbered females, with the majority of responders aged 55 and older. In all selected villages, the majority of respondents were cattle owners with 16 years or more of farming experience. Secondary school was the most frequently reported educational background in Phetole and Phiofu, while primary school was the most frequently reported in Phofu. According to the findings, the most common cattle breed owned in Phetole and Phofu was Nguni, while Afrikaner was the most common in Madietane, and the most common reason for keeping cattle was income in the study areas. The most frequently mentioned challenges in the areas are sickness (Soft hooves/or lumpy skin disease/or red water/or tick-borne disease), stock theft and disease, with Madietane having the highest mortality rate, followed by Phetole and Phofu. The most commonly reported perceived solutions were government assistance in terms of vaccinations/or veterinarians, dumping sites/or fixing water machines/or provide dams/or supplements, feed/or full-time patrollers to protect cattle from being stolen; and the purchase of cattle medicines. The majority of farmers in the selected villages can buy medicines for their cattle, while those who cannot say that the government assists them with vaccinations. As a result, it is concluded that there is a need for more knowledge and information on the subject.

## 1 Introduction

Global livestock production is predicted to more than double by 2050, from its current level [1,2]. Livestock production plays a crucial part in the natural economy of South Africa and beyond as a component of agriculture, providing food for both urban and rural inhabitants [3]. An estimated 80% of South Africa's agricultural land is suited for extensive grazing [4]. Extensive grazing is characterised by each animal grazing over a broad region with little labour and money required [5]. Cattle farmers in many rural communities often use this type of grazing, in which cattle only graze on natural rangeland for productivity [6,7].

Communal cattle farming is one of the world's oldest farming systems, practiced mostly by rural households in developing countries, particularly in Africa, and appears to be extremely resilient to any global economic crises to date [8]. Cattle are especially important in communities because they produce meat for a variety of purposes such as rituals, selling, and home consumption. According to Ref. [9], emergent and communal farmers provide 40% of cattle production in South Africa, despite sharing land for livestock grazing. In rural areas, cattle are habitually kept to supplement income from specialized activities. For example, purchasing building materials for a new home or paying for school tuition or a funeral [10]. Furthermore, cattle also provide meat for a number of uses, including ceremonies, selling, and domestic eating. However, communal farmers face a number of problems that prevent them from gaining a good yield from their cattle because they struggle to rise input costs such as cattle feed.

Cattle production in the subtropics is hampered by climatic stress, nutritional stress, and disease, as well as insufficient access to land and water, a lack of market channels, poor rangeland management, and a lack of feed resources [11]. As a result, vulnerability to these stressors has a significant impact on fertility, growth rate, and mortalities, all of which have a detrimental impact on cattle production [12]. Another key problem for communal farmers is the effectiveness of support services, particularly those related to animal health, nutrition, and the marketing of cattle and small stock [13]. As a result, government assistance for health issues is less effective because veterinarians and animal health professionals that assist communal farmers lack communication skills [14]. Therefore, the objective of this study was to investigate challenges and perception of farmers on cattle production in Ga- Matlala, Limpopo Province, South Africa.

## 2 Materials and methodology

### 2.1 Study site

**Fig. 1:**
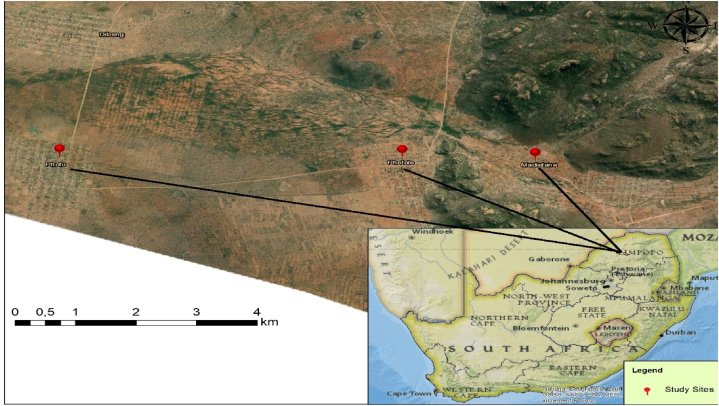
Map showing different villages (Phofu, Phetole, and Madietane) of the study area.

Source: Monkwe, TR. 2021. Map of Phofu, Phetole, and Madietane villages in Ga-Matlala area.

The research was carried out in three communal areas in the Capricorn District Municipality of South Africa's Limpopo province. Phofu, Phetole, and Madietane were the three communal areas (Fig. 1.) Polokwane Platea Bushveld is the vegetation type found in the three communal areas [15]. The area has a semi-arid environment, with an annual mean rainfall of 478 mm. The average temperature is 28.1 °C, with the highest temperature being 36.8 °C. The average minimum temperature during the dry winter season is 4.4 °C.

### 2.2 Population and sampling size

The survey was conducted with a total of 59 respondents in the three combined selected rural areas (Phofu, Phetole and Madietane). Participated respondents were 15 from Phetole, 15 from Phofu and 29 from Madietane areas. The small size and imbalance of farmers in the areas was because of Madietane had more cattle farmers than Phetole and Phofu and the unwillingness to continue with interviews. Each individual local Induna of each of the three villages (Phofu, Phetole and Madietane) was visited prior to data collection day to request permission to collect data and the letters of request were provided.

### 2.3 Sampling procedure and data collection

Snowball sampling technique also known as referral sampling was used to select 59 households that were interviewed in the three villages. The questionnaire was pre-tested before use in the survey to check its suitability. This was done to identify ambiguous questions. The respondents were interviewed at their homestead with pre-tested structured questionnaire. The interviews were conducted in the Sepedi vernacular by trained enumerators. The questionnaire consisted of four sections labelled as A, B, and C **Section A** captured data on household demographic information, **Section B** included herd size and management of cattle, **Section C** included farmers’ opinion on the challenges of cattle farming in the area.

### 2.4 Statistical analysis

Data were analysed using Statistical Package for the Social Sciences version 27 (SPSS, 2020). Chi-square (χ2) statistics were used to compare categorical variables between three villages. P- value was considered significant different at 95% interval (P < 0.05).

## 3 Results

### 3.1 Demographic information of the farmers

All demographic characteristics of the respondents (Table 1) did not differ significantly (P > 0.05) between the three villages. Males made up more than 60% of respondents in all three villages with the bulk of them being 55 years old. The vast majority of respondents were cattle owners with more than 16 years of farming experience. The majority of respondents in Photole and Mediatane had secondary school education, whereas the majority of respondents in Phofu (33.3%) had primary school education.

**Table 1:** Demographic information of the farmers.

Demographic information	Frequency (N)	Phetole Percentage (%)	Frequency (N)	Phofu Percentage (%)	Frequency (N)	Madietane Percentage (%)	Chi-square	*P*-value
**Gender**								
Male	13	86.67	10	66.67	20	68.97	1.96	0.38
Female	2	13.33	5	33.33	9	31.03		
**Age distribution**								
<25	0	0	0	0	0	0	8.72	0.19
25–34	1	6.67	1	6.67	7	24.14		
35–44	2	13.33	1	6.67	3	10.34		
45–54	0	0	1	6.67	5	17.24		
≥55	12	80.0	11	73.33		48.28		
**Position**								
Cattle owner	12	80.0	11	73.33	20	68.97	7.62	0.11
Herder		0	2	13.33	8	27.59		
Other (wife, sibling, children, neighbour etc.)	3	20.0	2	13.33	1	3.45		
**Farming experience (Years)**								
<2	1	6.67	2	13.33	1	3.45	6.99	0.54
3–5	3	20.0	2	13.33	7	24.14		
6–10	0	0	2	13.33	7	24.14		
11–15	2	13.33	2	13.33	3	10.34		
≥16	9	60	7	46.67	11	37.93		
**Educational background**								
Never attended	4	26.67	4	26.67	4	13.79	11.85	0.30
Primary school	4	26.67	5	33.33	5	17.24		
High school	6	40.0	4	26.67	15	51.72		
College diploma	0	0	2	13.33	2	6.90		
University degree	0	0	0	0	3	10.34		
Postgraduate qualification	1	6.67	0	0	0	0		

### 3.2 Number of cattle (N = 59), type of breeds and daily cattle management in the studied area

**Fig. 2:**
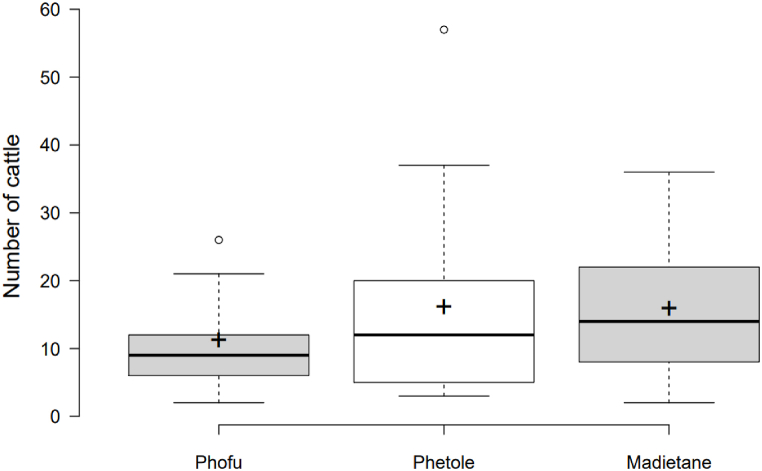
Number of cattle per village in the studied area. Note: The horizontal thick line is the median value, the plus sign (+) is the mean, the middle box indicates the inter-quantile range, whiskers represent values within 1.5 IQR of the lower and upper quartiles, and individual points show outlier values.

Fig. 2 depicts information on cattle distribution in the three studied communal areas. Although, there was no statistical significance (P > 0.05), Madietane had the most cattle kept, followed by Phetole and Phofu. Nguni was the most commonly owned breed in Phofu and Phetole, but the third most popular breed in Madiatane (P > 0.05). The most popular breeds in Madietane were Afrikaner, Nguni, and Afrikaner (P > 0.05) (Table 2). The daily cattle management differed significantly between the three villages (P < 0.05). The majority of respondents in Phofu reported that they release their cattle in the morning and return them late at night, with no provision for food or water, and that they rely on the community for water supply (Table 3). More than 50% of respondents in Phetole and Madiatane reported releasing cattle in the morning and returning late to provide them with feed, water, and medication.

**Table 2:** Summary of cattle breeds in the herds.

Breeds	Phofu		Phetole		Madietane		Chi-square	P-value
	Frequency (n)	Percentage (%)	Frequency (n)	Percentage (%)	Frequency (n)	Percentage (%)		
Afrikaner	3	10.3	4	26.7	8	27.6	23.66	0.10
Nguni	8	27.6	5	33.3	3	10.3		
Brahman	0	0	0	17	5	17.2		
Nguni and Afrikaner	3	20	4	26.7	8	27.6		
Nguni and brahman	0	0	2	13.3	2	6.9		
Nguni, Brahman and Afrikaner	0	0	0	0	1	3.4		
Cross (Brahman x Afrikaner)	1	3.4	0	0	1	3.4		
Cross (Nguni x Afrikaner)	0	0	0	0	1	3.4		
Cross (Nguni x Brahman x Afrikaner)	0	0	0	0	1	3.4

**Table 3:** Daily cattle management.

Cattle management	Phofu		Phetole		Madietane		Chi-square	P-value
	Frequency (N)	Percentage (%)	Frequency (N)	Percentage (%)	Frequency (N)	Percentage (%)		
1	11	73.3	7	46.7	8	27.6	9.08	0.04
2	3	20	8	53.3	19	65.5		
3	1	6.7	0	0	2	6.9		

**Note:** 1 = Released in the morning and returned late and depend on community water, 2 = Released in the morning and returned late and provide water/or medication/or food, 3 = Cattle stay at the mountains and returned home sometimes.

### 3.3 Reasons for keeping beef cattle and mortalities with reasons for cattle loss

**Fig. 3:**
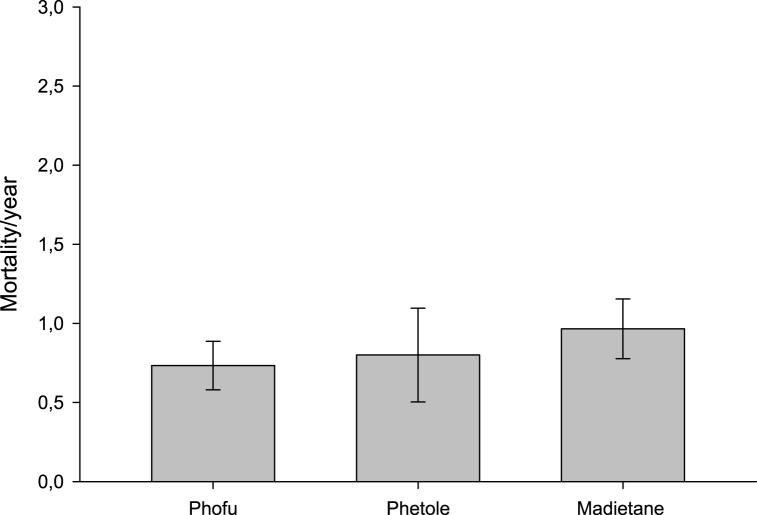
Mean number of mortalities of cattle occurred in a period of year in the studied communal areas.

Table 4 shows the respondents' perceptions on the reasons for keeping cattle. The reasons for keeping cattle did not differ significantly among the three villages (P > 0.05). In Phetole and Madiate, the main perceived reason for keeping cattle was income, followed by income and household consumption. While the most perceived reason for keeping cattle in Phofu were income and inheritance, followed by a love of farming. The annual mean number of cattle mortalities in the studied communal areas is depicted in Fig. 3. Madietane had the highest mortality rate, followed by Phetole and Phofu but there was no statistical significance among them. The causes of cattle loss in the three communal areas did not differ significantly (P > 0.05; Table 5). Diseases (Lumpy skin disease/or swollen hooves/or red water/or Bloating) were the most commonly perceived cause of cattle loss in all three villages. It was followed by starvation in Phofu and mysterious death in Phetole (P > 0.05).

**Table 4:** Reasons for keeping cattle in the three communal areas.

Reasons for keeping cattle	Phofu		Phetole		Madietane		Chi-square	P-value
	Frequency (N)	Percentage (%)	Frequency (N)	Percentage (%)	Frequency (N)	Percentage (%)		
Income	4	26.67	7	46.67	11	37.93	31.93	0.08
Inheritance	4	26.67	1	6.67	3	10.34		
Easy to handle	1	6.67	0	0	0	0		
Household consumption	2	13.33	0	0	0	0		
Status	0	0	1	6.67	1	3.45		
Draught	0	0	0	0	0	0		
Love for farming	3	20	0	0	2	6.90		
Income and household consumption	1	6.67	2	13.33	10	34.48		
Other	0	0	4	66.68	2	6.90		

**Note:** Other = (Such as Income and easy to handle; Income and household consumption; Household consumption and status; Easy to handle and household consumption; Income and love for farming).

**Table 5:** The reasons for cattle mortality in three studied communal areas.

Reasons for cattle mortality	Phofu		Phetole		Madietane		Chi-square	P-value
	Frequency (N)	Percentage (%)	Frequency (N)	Percentage (%)	Frequency (N)	Percentage (%)		
Diseases (Lumpy skin disease/or swollen hooves/or red water/or Bloating)	3	30	5	71.4	10	55.6	22.73	0.65
Killed by thieves	1	10	0	0	1	5.6		
Found dead (mysterious death)	1	10	1	14.3	1	5.6		
Starvation	2	20	0	0	0	0		
Plastic consumption	1	10	1	14.3	0	0		
Snake bite	1	10	0	0	0	0		
Poisonous plants	1	10	0	0	0	0		
Other	0	0	0	0	6	33.6		

**Note:** Other = (Such as Abnormalities from birth; Retained after birth; Diseases and snake bite; Diseases and found dead; Killed by thieves and snake bite; Diseases and killed by thieves).

### 3.4 Challenges in efficiency production, causes of challenges and perceived solutions

Table 6 shows the challenges that farmers face, and there were no statistically significant differences between villages (P > 0.05). Nevertheless, in Phetole and Phofu, stock theft was regarded as the most challenge, followed by disease. In Madiatene, disease and stock theft (33.03%) were perceived as the most significant challenge, followed by diseases (13.79%) and stock theft (13.79%). The shortage of herders was the most perceived cause of farmer issues in Phetole and Phofu, whereas it was the second most perceived cause in Madiatane, despite no statistical difference between communal areas (P > 0.05; Table 7). In Madiatane, however, the high unemployment rate was identified as the leading cause of problems. Table 8 shows farmers' perceived solutions to the problems they face. The perceived solutions to challenges differed significantly between villages (P < 0.05). The most common solution reported in Madiatane was government intervention, followed by the purchase of medicines and community unity. In Phetole, the majority of respondents reported no solutions to the challenges, which was closely followed by the purchase of feed and supplements and community unity. In Phofu, the major recorded solutions were government intervention, hiring more headers, and no solution to the problems.

**Table 6:** Challenges faced by the farmers that reduces the efficiency of production in the area.

Challenges	Phofu		Phetole		Madietane		Chi-square	P-value
	Frequency (N)	Percentage (%)	Frequency (N)	Percentage (%)	Frequency (N)	Percentage (%)		
Diseases	2	13.33	3	20	4	13.79	31.82	0.28
Stock theft	4	33.33	4	26.67	4	13.79		
Shortage of feed in winter	0	0	0	0	2	6.90		
Lack of water	0	0	0	0	1	3.45		
Lack of rainfall/drought	1	6.67	1	6.67	0	0		
Disease and shortage of food	0	0	1	6.67	0	0		
Stock theft and shortage of food	0	0	1	6.67	0	0		
Disease, stock theft and shortage of food	0	0	1	6.67	5	17.24		
Disease and stock theft	3	20	1	6.67	9	31.03		
Other	4	26.67	3	20	0	13.8		

**Note:** Other = (Such as Shortage of food, plastic consumption, and drought; Stock theft, thieves, and snake bites; Stock theft and snake bites; Disease, stock theft, and snake bites; Disease, stock theft, and snake bites; No challenges).

**Table 7:** The causes of challenges faced by the farmers in the studied communal areas.

Causes of challenges	Frequency (N)	Phetole Percentage (%)	Frequency (N)	Phofu Percentage (%)	Frequency (N)	Madietane Percentage (%)	Chi-square	P-value
High unemployment rate	1	6.67	2	13.33	3	10.34	67.45	0.43
Low rainfall	1	6.67	1	6.67	2	6.90		
No herder	2	13.33	3	20	2	6.90		
High alcohol consumption	1	6.67	0	0	0	0		
Low quality food	1	6.67	0	0	2	6.90		
Poisonous plants	1	6.67	0	0	0	0		
Other	8	53.35	9	60.02	18	65.54		

**Note:** Other = (Such as No dumping sites; Lack of vaccination, medication, and money; Surrounded by mountains and bushes; Taking bulls to auctions; Mixing of cattle herds; Broken water machines, lack of dams and rivers; Poisonous mosquitoes; Muddy soil; Injuries; Diarrhea etc.).

**Table 8:** Perceived solutions to the challenges.

Perceived solutions	Frequency (N)	Phetole Percentage (%)	Frequency (N)	Phofu Percentage (%)	Frequency (N)	Madietane Percentage (%)	Chi-square	P-value
Government intervention	1	6.67	3	20	16	55.17	36.65	0.02
Buy medicines	2	13.33	0	0	3	10.34		
Buy feed supplements and feeds	3	20	1	6.67	2	6.90		
Hiring more herders	0	0	3	20	1	3.45		
Unite as a community	3	20	3	20	3	10.34		
No solutions	6	40	3	20	0	0		
Other	2	13.34	2	13.34	4	13.8		

**Note:** Other = (Such as Buy medicines, feed supplements and feeds; Buy medicines and hiring more herders; Government intervention and hiring more herders; Government intervention and buy feed supplements; Buy our own bulls).

### 3.5 Government assistance

Table 9 shows explanations of government assistance. The explanations for government assistance were statistically significant (P < 0.05) among the villages. The majority of farmers in Phetole and Phofu reported receiving government assistance in terms of vaccination, whereas farmers in Madiatane reported receiving no assistance.

**Table 9:** Explanations on the government assistance.

Explanation	Frequency (N)	Phetole Percentage (%)	Frequency (N)	Phofu Percentage (%)	Frequency (N)	Madietane Percentage (%)	Chi-square	P-value
Yes, the government provides vaccinations only	8	53.33	6	40	9	31.03	22.07	0.02
Yes, but the government delivers after a long time	6	40	3	20	2	6.90		
No, the government is not helping	1	6.67	4	26.67	17	58.62		
Never applied for the government assistance	0	0	2	13.33	1	3.45		

## 4 Discussion

### 4.1 Demographic information of the farmers

In the current study, men made up the vast majority of respondents. This could be attributed to men's role in cattle management activities such as daily herding of cattle to grazing areas. Similar findings were reported by Ref. [16], who discovered that cattle farming remains primarily a male-dominated industry. Furthermore, the findings of this study were corroborated by Refs. [17,18,19], who discovered that men owned a large number of cattle, which they attribute to management challenges such as handling for treatment. This, however, contradicts the findings of [20] in Kampala, Uganda, who reported females as the dominated questioned farmers since males spend the majority of their time either conducting business or salaried job, allowing women to manage livestock on a daily basis.

The majority of farmers in this study were above the age of 54, indicating that elderly individuals are certainly involved in cattle farming in Ga- Matlala's areas with free communal grazing land. The findings of this study show elders have more time to care for cattle in rural areas than young people who reside in cities due to employment and school. Similar results were reported in Eastern Cape by Ref. [21] who stated that youth do not participate in livestock farming because they are more focus in urban areas than living in rural communities. However [21], found that the majority of respondents were between the ages of 31 and 50. According to Ref. [22], the high proportion of involvement of middle-aged farmers in agriculture was observed in North-West Province which confirms the low participation rate of youth in agricultural development. On the other hand [23], found that the majority of youth in Anambra State, Nigeria took part in crop production projects rather than livestock production. The vast majority of those who responded were cattle owners. This suggests that farmers value their farming as a major source of income. They take care of their own cattle rather than entrusting the management of their herd to others. Similar findings were reported by Ref. [24], who discovered that in most cases, family heads are in charge of running the day-to-day activities associated with livestock rearing. According to the above-mentioned author, a significant number of cattle owners also hire people to care for their cattle. However, it is unclear if the laborers are employed when the family head is present or absent to look after the cattle.

The majority of farmers in the three villages had 16 years or more of experience farming with cattle. This is consistent with the fact that older people are more involved in cattle farming than younger people. This result is in line with the findings of [21] who observed that the number of years of farming experience was above 15 years. Similar results were also reported by Ref. [25] who said that most of the farmers were above 21 years of farming experience of keeping cattle. These results contrast with the findings by Ref. [26] who reported 10 years livestock farming experience.

In the present study, high school was reported to be the highest educational level obtained by majority of the farmers in Phetole and Madietane while primary school was reported in Phofu. This result is a major advantage for the integration of communal area farmers into beef cattle value chain development projects. This finding was also unexpected in light of the number of older participants in the study area who depend on pensions for their income and who grew up in the apartheid era and had limited access to formal education [27]. These findings are similar with the reports of [17,28] who observed that more than 90% farmers participating in farming in Zimbabwe had secondary education level [28]. reported that majority of male had secondary education in Zimbabwe. The Food and Agriculture Organisation of the United Nations (FAO) emphasises that education, whether formal, non-formal or in the form of skills training, is very useful as develops the capacity of people to ensure food security [29]. However [30], argues that a level of education contributes to food security and poverty reduction since it opens up opportunities to improve on livelihood strategies.

### 4.2 Number of cattle (N = 59), type of breeds and daily cattle management in the studied area

In the present study, majority of the farmers reported large herd size in Madietane and Phetole. According to Vetter et al. (2020), larger herds suffer lower mortality rates, suggesting that owners of larger herds have better means to support their herds and owners of larger herds are wealthier and have more access to inputs and herding labour. This is comparable with studies that reported large herd size [31,32,33]. On the other hand, the average herd size to range from 5 to 10 cattle per household was reported by Refs. [34,35] with the purpose of primarily addressing needs for subsistence with limited use of technology in South Africa.

All of the farmers surveyed raised cattle that were either Afrikaner, Nguni, Brahman, crossbred, or a combination of the above breeds. However, most farmers in Phofu and Phetole indicated that they preferred to keep Nguni breeds, whereas in Madiatane, the most popular breeds were Afrikaner, Nguni, and Afrikaner. This could be owing to these breeds' ability to produce and their increasing tolerance to rural conditions [36]. found similar findings in Chief Albert Luthuli Local Municipality in Mpumalanga Province, stating that roughly 31% of farmers considered farming with the Nguni breed because to its high production capabilities. In contrast to the current findings [37], discovered that in the communal areas of the Eastern Cape Province of South Africa, farmers unintentionally farm with cross breeds due to unregulated mating as a result of insufficient fencing.

Cattle are normally freed in the morning and herded to the grazing areas, returned home later, and kraaled at night in the three villages. However, most farmers in Phetole and Madietane reported that even though they let cattle graze during the day and kraaled at night, they still provide them with feed, water, and medication when they return home later, whereas most farmers in Phetole reported that cattle rely on community water, so they do not provide them with food or water when they return home [38]. observed similar findings in the Vhembe District of Limpopo Province, where cattle are generally herded since there are no camps and kraaled every night throughout the year for fear of theft, traffic accidents, and crop damage prevention. Furthermore [39], stated that communal farmers rely on a variety of water sources, which vary based on location, season, and capacity.

### 4.3 Reasons for keeping beef cattle and mortalities with reasons for cattle loss

The study found no significant differences in the reasons for keeping cattle between the three villages. However, cattle serve multiple purposes in the communities studied. In Phetole and Madietane, income was the most important reason for having cattle, while in Phofu, income and inheritance were the most important reasons. Cattle farming is considered by communal farmers to be the most lucrative business. For instance, if a child is required to attend school and a school fee is required, the farmer cannot sell any other livestock than cattle because they believe that selling cattle will swiftly bring in a large sum of money and allow him to pay off the debts [36]. reported similar findings, stating that cattle farmers in Chief Albert Luthuli Local Municipality in Mpumalanga Province reported keeping beef cattle for sale and for their own consumption. These findings contrast with those of [35], who discovered that milk was the predominant reason for keeping cattle in two municipalities in the Eastern Cape. Farmers also acknowledged keeping beef cattle for inheritance. This meant that farmers may inherit cattle from their family members, such as parents, grandparents, or relatives, to bridge the gap and assure continuity [38]. found similar findings, stating that another reason communal farmers keep cattle is because they are inherited in Limpopo Province's Vhembe District. In the three communities studied, there were no significant variations in cattle mortality. However, Madietane was the area where the majority of cattle died, followed by Phetole and Phofu. These findings are consistent with those of [40], who found a 30% death rate in communal cattle farming. However, this finding contradicts with the findings of Makgatho (2006), who found a low cattle death rate in communal areas of the Odi district. The cattle mortality was mostly cause by diseases to the three studied villages [41]. found similar results, identifying diseases as the leading cause of cattle mortality in Western Kenya.

### 4.4 Challenges in efficiency production, causes of challenges and perceived solutions

The study discovered that stock theft was the most significant challenge in the three villages studied. Cattle are regularly stolen in these villages in the grazing areas because famers leave the cattle with no herders on the grazing fields. These findings are consistent with those of [42], who reported that cattle theft is an issue for farmers in the Northern KwaZulu-Natal Province of South Africa. The communal farmers reported that high unemployment leads the young people to steal cattle and selling them in order to make money since no herders on the fields. These findings are consistent with research that have linked high unemployment rates to cattle stealing [43,44] In Madietane, disease was also important challenge affecting the cattle production efficiency. Since cattle graze on communal rangelands, the increased presence of diseases could be attributed to inadequate nutrition or being infected by other sick animals. Similar results were also reported by Ref. [41] who highlighted disease as the major factor affecting cattle production. Farmers proposed government action in the form of job development, vaccinations, veterinarians, dumping sites, water machine repair, dam construction, feed, and full-time range patrollers to address the above-mentioned challenges. In the study by Ref. [36] in Chief Albert Luthuli Local Municipality in Mpumalanga Province, farmers suggested similar solution such as vaccination programmes as a solution to the problem of disease and ticks.

### 4.5 Government assistance

According to the findings of the study, the majority of farmers in Phetole and Phofu reported receiving government vaccination assistance. This indicates that the majority of farmers rely on government help to keep their cattle healthy. This is consistent with the findings of [24], who found that in South Africa's KwaZulu-Natal, Eastern Cape, Mpumalanga, North-West, and Free State provinces, most farmers indicate that animal health practitioners visit their animals on a frequent basis for vaccination.

## 5 Conclusions and recommendations

Findings from this study revealed that farmer's knowledge on issues such as herd size, cattle breeds in the herds, cattle management, reasons for keeping beef cattle and reasons for loss of cattle has been successfully evaluated in Ga-Matlala area, Limpopo South Africa. It was noted that the majority of respondents were males. Since most of the farmers are old people, it was expected that there will be the highest number of respondents who never attended school, however, high school and primary school were the highest educational background which means most of the farmers are literate and can be able to help themselves in most challenges related to beef cattle farming. Not only they depend on educational background, but farming experience also play a very important role in knowledge of beef cattle management, hence majority of beef cattle farmers have more than 16 years of farming experience.

The herd size was larger in the villages, this makes it difficult for them to manage their herds particularly when they are faced with challenges such as dry season when there is a shortage of feeds. Most of the farmers farm with Nguni and Afrikaner breeds, this is also an advantage since these breeds can survive harsh conditions, therefore can survive the dry season challenges. Highest mortality rate was reported in one village and disease was reported as the main cause of cattle mortalities, this can imply that most of the farmers that reported poor educational background may be those facing challenges and only less of those reported better educational background face these challenges. Another challenge that was mostly reported was cattle theft, this may be because of ignorance of farmers as they tend to leave cattle unattended at the grazing fields. However, solutions to the mentioned challenges need the government to assist. Regarding the daily cattle management, it is common in communal areas that livestock is released to grazing fields and brought home late, but provision of extra water and feeds is optional. Income was the most reason to keep cattle, this can be expected from each cattle farmer. Most of the farmers are able to buy medicines but others are not able to buy medicines. This means those that are not able to buy medicines rely on the government and if the government is not providing then their cattle production suffers because of disease. Moreover, farmers that are not able to buy medicines for their cattle have no money to buy or no knowledge on the medicines needed on the health of cattle, hence, they rely on the government. It is thus concluded that there is a need for more knowledge and information on the overall cattle management which will assist by reducing mortality rates and overcome several difficulties they reported, particularly with regard to disease management and theft for enhancing productivity in the communal areas.

### 5.1 Recommendations

1 Encourage knowledge creation and sharing among farmers

Decentralized information management and sharing of appropriate knowledge using new technologies such web-based applications like management database systems is encouraged. Such systems encourage farmers to identify, share and prioritise their problems and needs but most importantly, to seek ways of solving their problems within their community rather than waiting for the government's extension service. The database will also, not only serve as an information sharing platform but for record keeping as it is capable of producing documented decisions based on accessible and reproducible knowledge. For sustainability of the system, farmers should be asked to pay an annual fee for the maintenance and upgrading of the systems.

2 Public and private sector support for infrastructural development

It is important for the government to strengthen public-private partnership in financing the establishment of production and marketing infrastructure in the communal farming communities. Multi-stakeholder cattle farming development forums targeting emerging and communal farmers at local and district municipalities as well as provincial level could be a good way to foster these linkages or partnerships. Such forums should then be used in identifying the appropriate infrastructure and proposed locations for the benefit of the farmers. Investment in production infrastructure will improve productivity and cushion some of the challenges reported in this study. These include theft, disease and predators. On the other hand, availability of marketing infrastructure will significantly improve the ability of farmers to reduce transaction costs, improve market access and access to market information.

## Author contribution statement

Thapelo Rosina Monkwe: Performed the experiments; Analysed and interpreted data; Wrote the paper.

Masibonge Gxasheka: Conceived and designed the experiment; Contributed reagents, materials, analysis tools or data.

Busisiwe Gunya: Conceived and designed the experiments; Analysed and interpreted the data; Wrote the paper.

## Funding statement

Thapelo Rosina Monkwe was supported by National Research Foundation [MND190909475603].

## Data availability statement

Data will be made available on request.

## Declaration of interest’s statement

The authors declare no competing interests.
