# Supplementary material for: Challenges and perception of communal farmers on cattle production in Ga- Matlala, Limpopo Province, South Africa
Source: Heliyon. 2023 Mar 5;9(3):e14190. doi: 10.1016/j.heliyon.2023.e14190 (PMC10015193; doi:10.1016/j.heliyon.2023.e14190)
Supplement: Multimedia component 1 [file mmc1.docx]

## Appendix A: Questionnaire

# **QUESTIONNAIRE FOR COMMUNAL BEEF CATTLE FARMERS**

*All the information provided here will be treated as strictly exclusive. Data gathered by this questionnaire will be used only for the purpose of this intended evaluation and nothing else. Personal and socio-economic information of respondents will be kept confidential and no mention of names shall be made in the final report that shall be compiled. For purposes of report, it is hereby required that consent is given through signing the declaration below by the respondent before the beginning of the application.*

**1. SECTION A: *Demographic Information***

| *Names:* |  | | | | | | | | | |
| --- | --- | --- | --- | --- | --- | --- | --- | --- | --- | --- |
| *Contact Number:* |  | | | | | | | | | |
| *Village name:* |  | | | | | | | | | |
| ***Please mark the appropriate answer with an X in the box of the table provided*** | | | | | | | | | | |
| *What is your gender* | *Male:* | | | | | | | *Female:* | | |
| *Indicate your age group* | *24 years and younger* | | | *25 to 34 years* | | *35 to 44 years* | | | *45 to 54 years* | *55 years and over* |
| *Indicate your Position* | *Cattle farmer*  *(Owner):* | | | | *Cattle herder* | | | *Other*  *(Specify):* | | |
| *Indicate your farming experience* | *Less than 2 years* | | *3 to 5 years* | | | | *6 to 10 years* | | *11 to 15 years* | *16 years and over* |
| *Indicate your educational background* | *Never attended* | *Primary school* | | | *High School* | | *College Diploma* | | *University Degree* | *Post-Graduate qualification* |

**2. SECTION B: *Herd size and management***

- 1. *How many cattle are under your care (including calves)?*

|  |
| --- |
|  |

- 1. *Which beef cattle breeds are you farming with?*

|  |
| --- |
|  |

- 1. *How many cattle die over a period of 12 months (Mortality rate)?*

|  |
| --- |
|  |

- 1. *What are the causes of mortalities mentioned in 2.3?*

|  |
| --- |
|  |
|  |
|  |
|  |
|  |
|  |
|  |

- 1. *How do you manage your cattle daily? Please explain.*

|  |
| --- |
|  |
|  |
|  |
|  |

**3. SECTION C: *Farmers’* *opinions on the challenges of cattle farming in the area*.**

- 1. *Why did you decide to farm with cattle?*

|  |
| --- |
|  |
|  |
|  |

- 1. *What are the challenges faced by beef cattle farmers that reduces the efficiency of production your area?*

|  |
| --- |
|  |
|  |
|  |
|  |
|  |

- 1. *What are the reasons/causes of the problems listed in number 3.2?*

|  |
| --- |
|  |
|  |
|  |
|  |
|  |
|  |
|  |

- 1. *What are perceived solutions to the challenges listed in number 3.3?*

|  |
| --- |
|  |
|  |
|  |
|  |
|  |
|  |
|  |
|  |
|  |
|  |

- 1. *Do you buy medicine/drugs for your animals? If yes, list them, if no provide a reason.*

|  |
| --- |
|  |
|  |
|  |
|  |
|  |
|  |

- 1. *Do you get assistance from the government? Please explain.*

|  |
| --- |
|  |
|  |
|  |
|  |
|  |
|  |
|  |

**************************** End of Survey *************************
